# Mechanisms of Change in a Self-Help Parenting Program for Child Behavioral Difficulties: the Role of Unsupportive Parenting

**DOI:** 10.1007/s10802-025-01378-y

**Published:** 2025-11-13

**Authors:** Suzanne R.C. de Jong-Arts, Barbara J. van den Hoofdakker, Jos W.R. Twisk, Jaap Oosterlaan, Marjolein Luman

**Affiliations:** 1 Department of Clinical-, Neuro‐, and Developmental Psychology, Amsterdam Public Health Research Institute, Vrije Universiteit Amsterdam, Amsterdam, The Netherlands; 2 Department of Psychiatry, University Medical Center Groningen, University of Groningen, Groningen, The Netherlands; 3 The Research School of Behavioural and Cognitive Neurosciences, University of Groningen, Groningen, The Netherlands; 4 Accare Child Study Center, Groningen, The Netherlands; 5 Department of Clinical Psychology and Experimental Psychopathology, University of Groningen, Groningen, The Netherlands; 6 Department of Epidemiology and Data Science, Amsterdam University Medical Centers, Amsterdam, the Netherlands; 7 Department of Pediatrics, Emma Neuroscience Group, Emma Children’s Hospital, Amsterdam UMC, Amsterdam Reproduction & Development research institute, University of Amsterdam, Amsterdam, The Netherlands; 8 Levvel, specialists in youth and family care, Amsterdam, The Netherlands

**Keywords:** Behavioral parent training (BPT), Behavioral difficulties, Self-help parenting program, Mediator, Parenting practices

## Abstract

Self-help parenting programs that are aimed at reducing child behavioral difficulties may be effective and accessible alternatives to face-to-face parenting programs. Insight into the mechanisms of change for such programs is important to enhance their effectiveness. The goal of this study was to examine whether changes in supportive and unsupportive parenting (measured by the Parenting Practices Interview; PPI) and parenting sense of competence (measured by the Parenting Sense of Competence scale; PSOC) mediated the effects of a self-help parenting program on child behavioral difficulties. A total of 110 families were randomized to a 15-week self-help program or waitlist. Outcomes and mediators were measured at pre-, mid- and post-measurement. Outcomes were parent-rated child behavioral difficulties, assessed by the Eyberg Child Behavior Inventory, Intensity Scale (ECBI-I), and ecologically momentary assessments (EMA) of child behavior problems. Results showed that reductions in unsupportive parenting significantly mediated reductions in child behavioral difficulties assessed with the ECBI-I. We did not find this mediating effect for the EMA. Supportive parenting and parenting sense of competence did not mediate changes in child behavioral difficulties. Although future research into self-help parent training should provide insight into possible reciprocal interactions between changes in parents and children, reducing unsupportive parenting practices may play a key role in reducing child behavioral difficulties.

**Supplementary Information:**

The online version contains supplementary material available at 10.1007/s10802-025-01378-y.

## Introduction

Behavioral parent training is an effective treatment for reducing child behavioral difficulties, including attention problems, hyperactive, impulsive and/or oppositional behaviors (Hornstra et al., 2023; Mingebach et al., 2018). However, access to these programs can be limited (Koerting et al., 2013) and self-help parenting programs may provide a more accessible and feasible alternative. Previous studies showed that self-help programs yield small (Thongseiratch et al., 2020) to large (Tarver et al., 2014) effects on parent-rated child behavioral difficulties (see also de Jong et al., 2023) and small to moderate effects on parenting behavior and parental well-being (Tarver et al., 2014; see also de Jong et al., preprint 2024). These results are comparable to the effects reported in face-to-face programs, which show moderate improvements in child behavior (Mingebach et al., 2018) and small to moderate improvements in parental outcomes (Dekkers et al., 2022). Despite the demonstrated efficacy of parenting programs little is known about the mechanisms underlying child behavioral changes through parent training, particularly with regard to self-help programs. Insight into these underlying mechanisms of children’s behavioral changes through parent training may help to enhance the effectiveness of parenting interventions.

One of the most plausible mechanisms underlying improvements in child behavior following behavioral parent training are changes in parenting practices. Parenting programs are specifically designed to promote supportive parenting practices and reduce unsupportive ones. This would interrupt the coercive cycle in which both the parent’s and child’s negative behaviors are reinforced (Van der Oord & Tripp, 2020). However, research examining parenting as mediator between parent training and child behavioral changes after parent training has yielded mixed results. A review of 25 studies on the mediating role of parenting practices in face-to-face parenting interventions for children with behavioral difficulties (oppositional defiant or conduct disorder symptoms) between 2 and 18 years old (Forehand et al., 2014) took positive parenting, negative parenting, monitoring/supervision, and discipline (appropriate versus inappropriate) as candidate mediators into account. Positive parenting and monitoring (both supportive parenting techniques) acted as a mediator of changes in child behavioral difficulties in 45% and 10% of the included studies, respectively. Negative parenting (unsupportive parenting) mediated changes in child behavioral difficulties in only 26% of the studies. Additionally a shift from inappropriate to appropriate discipline (i.e., a shift from unsupportive to supportive parenting), was found to act as a mediator in 55% of the included studies.

Two studies have investigated mediators regarding parent training for children with attention-deficit/hyperactivity disorder (ADHD). The first study showed that reductions in children’s ADHD behaviors after a parenting program were mediated by reductions in unsupportive parenting, and not by increases in supportive parenting (Chronis-Tuscano et al., 2011). The second study on mediators of a multimodal behavioral therapy (including parent training) also showed that reductions in unsupportive parenting mediated improvements in ADHD behaviors, while this was not the case for supportive parenting. However, this mediating effect was only present in a group of children that received a combination of medication and multimodal behavioral therapy, and not in a group that was given multimodal behavioral therapy only (Hinshaw et al., 2007).

Another parental factor that may underly change in child behavioral difficulties following parent training is parenting sense of competence (Tarver et al., 2014). Parents with a low sense of competence might have difficulties in consistently using supportive parenting techniques if their child’s behavior becomes more challenging (Jones & Prinz, 2005). This can contribute to a negative coercive cycle, in which parents give up, followed by an increase in child behavioral difficulties. Subsequently, parents’ believes regarding the quality of their parenting may be confirmed (Jones & Prinz, 2005). Studies assessing parenting sense of competence as a putative mediator in parent training are scarce and limited to face-to-face parenting interventions. One of these studies showed that increased parental self-efficacy mediated reductions in ADHD behaviors and conduct problems among 3 to 8 year old children following parent training (Rimestad et al., 2020). Another one demonstrated sequential mediation of parenting sense of competence and supportive parenting, in a multi-systemic intervention including parent training for adolescents with behavioral difficulties: an increase in parenting sense of competence led to an increase in supportive parenting, which in turn led to improvements in child behavioral difficulties (Deković et al., 2012).

The mixed findings regarding supportive or unsupportive parenting as mediators (Forehand et al., 2014) maybe partly explained by the heterogeneity across studies. Previous studies into mediators in parent training programs differed in the type of parenting intervention they examined. Some studies examined mediators in face-to-face parent training only (e.g. Dishion et al., 2008), while others investigated multimodal behavioral interventions that included a combination of parent, child and teacher training (e.g., Hinshaw, 2007), where different mechanisms of effectiveness may play a role. Studies also varied in their focus on prevention (e.g., Lochman & Wells, 2002) versus treatment (e.g., Hagen et al., 2011), in which different mechanisms of change may be involved (e.g., children may show more severe child behavioral difficulties in intervention versus prevention studies).

Previous studies also differed in the type of methodologies to examine mediators of changes through parent training (Forehand et al., 2014). Some researchers consider it a prerequisite that the mediator precedes the outcome (Kazdin, 2007) and thus, that multiple assessments over time are needed in order to study mediation. However, in the context of parent training, changes in child behavior are typically expected to occur shortly after parenting practices are implemented, rather than following a (prolonged) delay. For example, Hornstra and colleagues found that behavioral problems decreased immediately after just two sessions of parent training, and remained stable thereafter (Hornstra et al., 2023). For this reason, it may be more appropriate to assess the mediator and outcome simultaneously (see for example Bjørknes et al., 2012; Gardner et al., 2006) instead of having a large delay between mediator and outcome (see for example Compas et al., 2010; Somech & Elizur, 2012), which may explain differences in type and strength of mediators obtained.

To date, only one study focused on mediators of change in a self-help parenting program targeting child behavioral difficulties in a group of children with ADHD. That study showed that a reduction of unsupportive parenting mediated children’s functional impairment and oppositional behavior, but not in their ADHD symptoms (Dose et al., 2021). Supportive parenting did not mediate child outcomes.

The aim of the current study was to study putative mediators of change in child behavioral difficulties after a 15-week self-help parenting program which was shown to effectively reduce child behavioral problems in a previous study (de Jong et al., 2023). Based on previous studies (mostly concerning face-to-face parenting programs), we hypothesized that supportive parenting, unsupportive parenting and parenting sense of competence would mediate the effect of self-help parent training on child behavioral difficulties. This was examined through a mediation analysis within the framework of a randomized controlled trial, looking at the mediator and outcome simultaneously at both the mid-measurement and the post-measurement.

## Method

### Design

We conducted a non-masked, parallel, randomized controlled trial with two active conditions – self-help parent training with or without bi-weekly telephonic support – and a waitlist control condition. As treatment effects did not significantly differ between the two active conditions (support versus no support 0.442 < *p* <.887; see de Jong et al., 2023), they were combined into a single intervention condition for the current study. Participants in the intervention condition completed the parenting program over a 15-week period. Assessments were conducted at three time points, pre-measurement at week 0, mid-measurement at week 8 (only for some measurements), and post-measurement at week 15. By adding an assessment in between our pre- and post-measurement, we could use both the mid-measurement and post-measurement in the mediation analysis, to increase statistical power and reliability of the mediator estimation. All measurements were parent-reported. Participants in the waitlist condition were permitted to seek care as needed during the waiting period and received access to the program after the 15-week intervention period.

## Participants

Parents were recruited through local child support facilities, social media, schools, and the Dutch parent association for children with developmental problems (i.e., “Oudervereniging Balans”). Eligibility criteria included (1) the parent was seeking help for their child’s behavioral difficulties; (2) the child was between 4 and 12 years old; (3) the child exhibited (sub)clinical levels of behavioral difficulties at home, which was determined by (a) a score above the 80th percentile (i.e., a score > 6 for children aged 4 or 5 years, or a score > 7 for children aged 6 to 12 years) on the subscale Externalizing Behavior of the Strengths and Difficulties Questionnaire (SDQ; Van Widenfelt et al., 2003), and (b) at least three symptoms of ADHD (hyperactivity/impulsivity and/or inattention) and/or at least two symptoms of oppositional defiant disorder (ODD) according to the Diagnostic Interview Schedule for Children, fourth edition (DISC-IV; Association, 2013; Park et al., 2015; Shaffer et al., 2004); (4) the child experienced functional impairment due to the reported behavioral difficulties, as determined by a rating *≥* 4 on one of the domains of the Impairment Rating Scale (IRS; Fabiano et al., 2006). For a detailed description of the screening measures (SDQ, DISC-IV and IRS), see (de Jong et al., 2023). Exclusion criteria were: (1) the parent currently or recently received parent training (within the preceding six months); (2) the parent already planned to start parent training or counseling focused on their child’s behavioral difficulties in the study period; (3) the child initiated using psychotropic medication or changed dosage within three months prior to the study; (4) parents’ reading ability was insufficient to fully understand the program materials; (5) the family planned a vacation away from home for more than two weeks during the study period.

## Materials

### Demographic Characteristics

Demographic characteristics (i.e., child’s age, sex and medication use, parent’s age and sex, and consumption of other care during the study) were assessed with parents using a custom made questionnaire. Intervention engagement (number of completed modules) was monitored using a custom-made parent-report questionnaire, which was distributed to parents every three weeks, and with data from the online program. Parents indicated which modules they worked on in the past three weeks (i.e. “Which module(s) have you read or worked on in the online program in the past three weeks (since the last time you filled out this questionnaire?”) and to what extend they had completed the manual and online program of those modules on a scale of 0 (not at all) to 3 (completely). Scores *≥* 2 were interpreted as sufficient engagement. Online program completion was determined by combining data from the program platform and the parent-report questionnaire, using the higher of the two as the completion level. Module 10 was not taken into account in program completion, as it was optional. Program fidelity regarding the use of techniques was measured using a parent-report questionnaire, where they indicated for each of the techniques if and to what extend they applied the technique in the past three weeks at home on a scale from 0 to 3 (not at all, less than 50% of the days, more than 50% of the days, every day), for example “Have you practiced/applied the techniques covered in Module 6 (Clear Rules and Assignments) at home? This refers to designing and applying rules as well as giving and monitoring instructions.” Using the techniques more than 50% of the days was considered sufficient. Lastly, 15 weeks after program completion, parents were asked which behavioral techniques they continued to use at least once per week (0 = no, 1 = yes).

### Outcome Measures

Child behavioral difficulties over the past two weeks were assessed with the Intensity scale of the Eyberg Child Behavior Inventory (ECBI-I; Eyberg, 1999). This scale consists of 36 items that describe specific behavioral difficulties, such as “refuses household chores”, of which parents rated the frequency on a 7-point Likert scale (1 = never, 7 = always). The ECBI-I has demonstrated robust psychometric properties in previous research, including excellent internal consistency (α = 0.92), good test-retest reliability (α = 0.84), and favorable convergent, divergent, and discriminative validity (Abrahamse et al., 2015). In the present study, during baseline measurement, the Intensity scale showed good reliability (α = 0.81).

In addition, to evaluate child behavioral difficulties that were specifically targeted in the intervention, Ecological Momentary Assessment was used (EMA; hereafter referred to as daily measurements). Over four consecutive school days, parents participated in three-minute phone calls, where they reported whether their child exhibited 14 specific behavioral difficulties, within the past 24 hour. The 14 behaviors were derived from a modified list of target behaviors (see Van Den Hoofdakker et al., 2007) that corresponded to the behaviors that were actually targeted in the parenting program. For each behavior, parents reported the occurrence, and if present, rated severity on a six-point Likert scale (ranging from 0 = did not occur, 1 = not severe to 5 = very severe). Examples of these behaviors included noncompliance and hyperactivity/restlessness (see Online Resource 1 for the full item list). The phone calls were protocolized and conducted by trained research assistants, who were masked to study condition. The outcome measure (daily measurements) was the mean severity score averaged over the four days. When two or more measurements were missed, they were rescheduled for the following week, and if unsuccessful, considered as missing data. Participants missing data for three or more days were excluded for that measurement week. Days where parents reported their child being ill or where parents had minimal contact with the child, were omitted from the analyses. Internal consistency of the daily phone calls during baseline in the current sample was good (*α* = 0.83).

### Putative Mediators

To assess supportive and unsupportive parenting practices, the Parenting Practice Interview questionnaire (PPI; Webster-Stratton, 2001) was used. Supportive parenting techniques include praise, providing clear rules and instructions, ignoring mild unwanted behavior and effectively setting limits (e.g., Goagoses et al., 2023; O’Brien et al., 2023). Unsupportive parenting includes harsh or rejective parenting, inconsistent parenting, and physical punishment (e.g., Goagoses et al., 2023; O’Brien et al., 2023). On the PPI, parents rate the likelihood of using specific parenting practices in response to certain behaviors of their child (for example “misbehaving”), on a 7-point Likert scale. Examples of practices include “giving the child a brief time out”, “scream towards the child” or “praising the child”. For the purpose of this study, two subscales were created: PPI positive and PPI negative consistent with prior research combining scales (e.g., Broderick & Carroll, 2008; Brotman et al., 2003; Weeland et al., 2017). Supportive parenting (PPI Positive) was measured by taking the average of the mean scores on the subscales Appropriate Discipline (12 items), Positive Verbal Discipline (9 items), Praise and Incentives (11 items), and Clear Expectations (6 items). To assess unsupportive parenting (PPI Negative), the mean score on the 15-item subscale Harsh and Inconsistent Discipline was used. The subscales Monitoring and Physical Punishment scales were omitted, as monitoring was not targeted in the parenting program, and physical punishment rarely occurred in our sample (*M* = 1.23 out of 7; *SD* = 0.39). The PPI Positive and PPI Negative scores may range from 1 to 7, with higher scores indicating higher supportive or unsupportive parenting practices, respectively. Although the validity and reliability of the PPI have not been examined in prior research, the current study showed, at baseline, good internal consistency (*α* = 0.83) for PPI positive, and acceptable for PPI Negative (*α* = 0.79).

To assess parenting sense of competence, the Parenting Sense of Competence Scale (PSOC; Johnston & Mash, 1989) was utilized. The PSOC comprises 16 statements, such as “Being a parent is manageable, and any problems are easily solved” which are rated by parents on a 6-point Likert scale (1 = strongly agree, 6 = strongly disagree). Total score is derived by adding scores on all items, with higher scores indicating a greater sense of competence in parenting. Previous studies reported acceptable to good concurrent validity and acceptable internal consistency (α = 0.79) of the total PSOC score (Johnston & Mash, 1989; Ohan et al., 2000). In the current sample, the PSOC demonstrated good internal consistency at baseline (*α* = 0.82).

### Intervention

The intervention comprises a manual and an online program, designed to teach parents common behavioral techniques (see below) to modify their child’s behavior. The program consists of 11 modules, which are ideally completed in 15 weeks. The program begins with two modules of psycho-education followed by eight modules covering specific behavioral techniques and concluding by a module summarizing the content, and addressing relapse prevention. Each module starts with reading a chapter in the manual, describing the technique of the module. Subsequently, the online program assesses parents’ comprehension and contains exercises guiding parents with implementing the behavioral techniques. Throughout the program, parents regularly rate the severity of their child’s behavioral difficulties and their own perceived efficacy in raising their child. The online program is individualized, focusing only on behavior and techniques that are relevant for the child’s and parents’ needs.

The techniques that were covered by the program were meant to stimulate supportive parenting practices and to find alternatives for unsupportive parenting practices. Two modules address providing supportive feedback and other rewards to the child, to reinforce desirable behavior. One module focuses on enhancing positive parent-child interaction through play. Two modules are aimed at providing clarity and predictability for the child: parents learn how to structure the child’s environment and routine and how to provide clear rules and instructions. Two models address managing challenging behavior of the child through selective ignoring and using mild consequences for unwanted behavior, with guidance on appropriate application. Finally, an optional module covers how to cope with temper tantrums.

### Procedure

Data were collected between February 2019 and March 2021. Parents could participate either alone or together with their partner, if being part of the same household. The parent most engaged with the intervention was designated the primary respondent for questionnaire completion (optionally in collaboration with their partner, if being part of the same household). When meeting inclusion criteria, parents were randomly allocated to one of the three conditions using a computer-generated number list, by an author who did not have any contact with the participants (ML). Medical ethical approval of this study was waived for the medical research with human subjects act (WMO, 2021) by the Medical Ethical Committee of the Vrije Universiteit Medical Centre Amsterdam (#2018.421). The trial was registered in the Dutch register of research with human participants (#26832). To report on the study, CONSORT guidelines were followed (Schulz et al., 2010).

### Data-Analyses

**Fig. 1:**
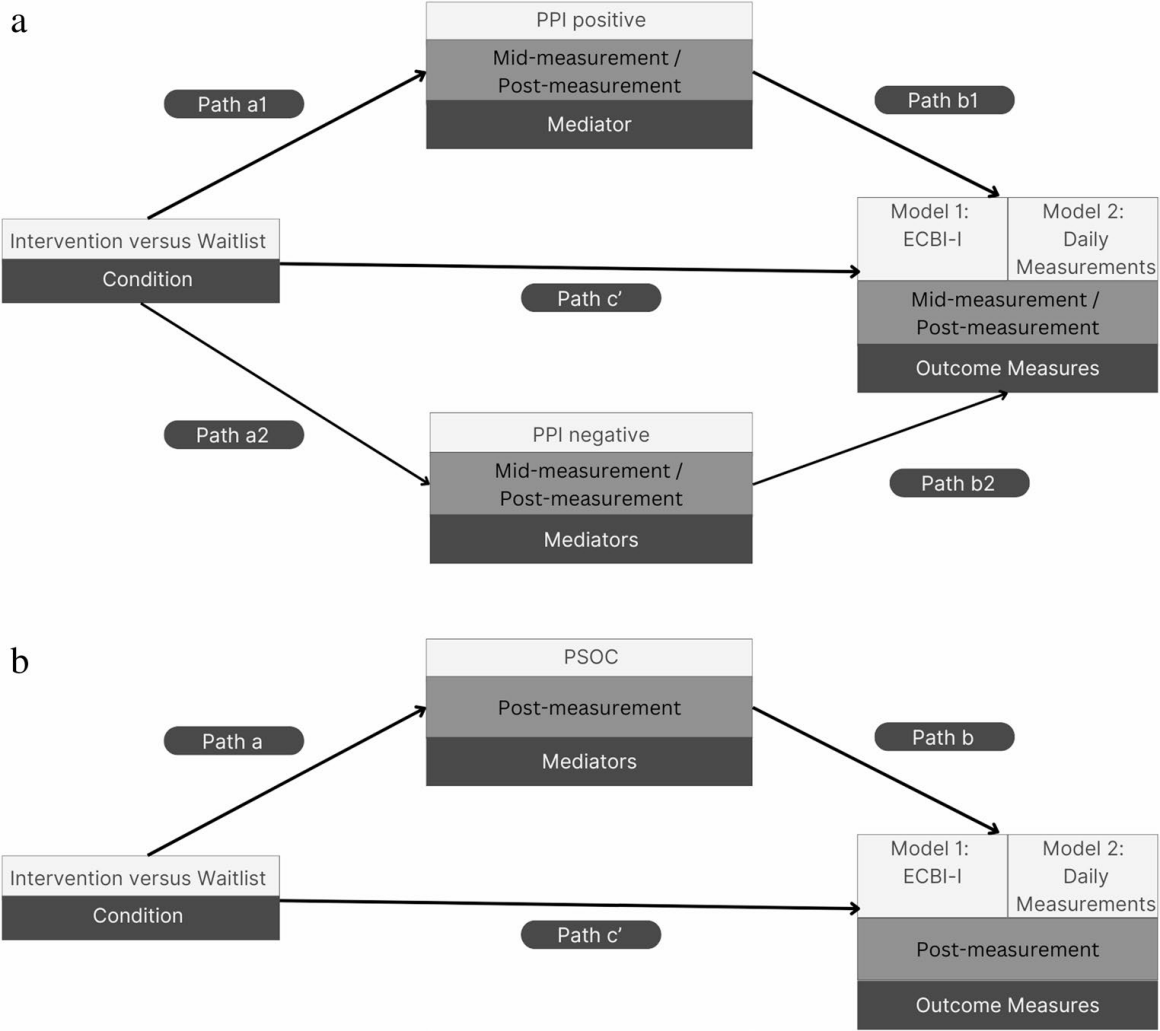
Visual Representation of the Mediation Models. **a. **PPI as Putative Mediator. **b. **PSOC as Putative Mediator. Note. Baseline levels of mediator variables and outcome variables were added as covariates with model 1 and 2 as separate models; ECBI-I = Eyberg Child and Behavior Inventory, Intensity Scale; PPI = Parenting Practices Interview; PSOC = Parenting Sense of Competence Scale

Analyses were based on intention-to-treat. Data were analyzed with SPSS (IBM Corp, 2016) and STATA (StataCorp, 2023). Normality of the data was visually inspected and extreme cases (deviating more than three SD from the mean) were winsorized (Blaine, 2018). Mediation analyses were performed using gsem in STATA (StataCorp, 2023). A visual representation of the mediation models is shown in Fig. 1. Parenting behaviors were analyzed using, a multilevel longitudinal regression analysis, with condition (intervention versus waitlist) as independent variable, PPI positive and PPI negative (both mid- and post-measurement) as putative mediators, and child behavioral difficulties (both mid- and post-measurement) measured by ECBI-I and daily measurements as outcomes. Separate models were estimated for both measures of child behavioral difficulties. PPI positive and PPI negative were entered simultaneously in the longitudinal mixed model mediation model. The model contained observations (level 1) nested within participants (level 2), and time (mid-study measurement and post-measurement) was defined as within subject fixed variable. Mediator and outcome measures were taken into account at the same time point in our analyses. For example, we examined the relationship between mid-measurements supportive parenting and mid-measurement child behavioral problems. To adjust for possible baseline differences in outcome measures between conditions, baseline measurement (pre-study measurement) of the mediators and outcome measures were added as fixed factor in the model (Twisk et al., 2018).

Due to PSOC only being assessed at pre- and post-, and not at mid-measurement, longitudinal mediation modeling was not possible. We analyzed the potential mediating role of PSOC with a simple mediation model with condition as independent variable, PSOC as putative mediator and ECBI-I and daily measurements as dependent variables in separate models, adding baseline measurements as fixed factor.

## Results

### Descriptives

The average age of the primary parents was 40.56 years (*SD* = 5.31) and 101 (91.8%) of them were female. The average age of the children was 8.17 years (*SD* = 2.29) and 80 (72.7%) of them were boys. Parents completed 7.64 out of 10 modules (*SD* = 3.06) of the manual and 7.71 out of 10 modules (*SD* = 2.82) of the online program (engagement measures). Parents indicated to have implemented an average of 5.45 out of 8 (*SD* = 2.26) of the behavioral techniques and that they kept using an average of 3.94 techniques (*SD* = 1.92) after 15 weeks (fidelity measures).

Baseline demographic characteristics of participants in the intervention and waitlist condition are presented in Table 1. There were no significant differences between groups regarding parent age and sex, child age and sex, severity of child behavioral difficulties and impairment, and medication use. Furthermore, there were no differences between the conditions in consumption of additional care during the study (see de Jong et al., 2023).

**Table 1:** Characteristics of Primary Parents and Children in the Intervention and Waitlist Condition

	Intervention condition (*n* = 74)	Waitlist condition (*n* = 36)
Baseline primary parent characteristics		
Age in years, *M (SD)*	40.68 (4.88)	40.32 (6.21)
Sex, females, *n* (%)	68 (91.9)	33 (91.7)
Baseline child characteristics		
Age in years, *M (SD)*	8.24 (2.32)	8.14 (2.26)
Sex, boys, *n* (%)	50 (67.6)	30 (83.3)
SDQ Externalizing Scale, *M (SD)*^a^	12.01 (2.24)	11.14 (2.17)
ADHD according to DISC-IV ^b^, *n* (%) Clinical Subclinical Nonclinical	55 (74.3) 18 (24.3) 1 (1.4)	31 (86.1) 5 (13.9) 0 (0)
ODD according to DISC-IV^c^, *n* (%)		
Clinical	57 (77.0)	31 (86.1)
Subclinical	14 (18.9)	3 (8.3)
Non-clinical	3 (4.1)	2 (5.6)
Impairment, *M (SD)*^d^	6.23 (1.56)	5.93 (1.38)
Psychotropic medication (y/n), *n* (%)^e^	16 (21.6)	12 (33.3)

There were no significant differences between conditions on any of the characteristics; *ADHD* attention-deficit/hyperactivity disorder, *DISC-IV *Diagnostic Interview Schedule for Children, fourth edition, *ODD *oppositional defiant disorder, *SDQ* Strength and Difficulties Questionnaire; ^a^ Range: 0–20; ^b^A clinical score was defined as at least six DISC-IV ADHD symptoms and a subclinical score as at least three DISC-_IV ADHD symptoms; ^c^A clinical score was defined as at least four DISC-IV ODD symptoms and a subclinical score as at least two DISC-IV ODD symptoms; ^d^Range: 0–10 on the Impairment Rating Scale (IRS); ^e^Type of medication in the intervention condition: n = 13 methylphenidate, n = 1 dexamphetamine, n = 1 lisdexamphetamine, n = 1 aripiprazole; ^e^Type of medication in the waitlist condition: n = 12 methylphenidate

Our previous study showed that the intervention significantly reduced behavioral problems, as compared to waitlist, see online resources Table S1.

### Mediation Analyses

Two cases (ECBI post-measurement and PPI positive mid-measurement) exceeded three standard deviations from the mean and were winsorized. Means and standard deviations of the mediators and outcome measures across time points in both conditions are shown in Table 2. Table 3 shows the results of the mediation analyses. PPI negative significantly mediated the relationship between condition (intervention versus waitlist) and ECBI-I score: After following the intervention, participants had significantly lower ECBI-I scores than after waitlist, and reductions in PPI negative were related to reductions in ECBI-I (partial mediation percentage: 19%). In contrast, PPI negative did not mediate the relationship between condition and daily measurements. All other mediation results (PPI negative as a mediator for daily measurements, and PPI positive and PSOC for both ECBI-I and daily measurements) were non-significant.

**Table 2:** Means and Standard Deviations of Mediators and Outcome Measures at Pre-, Mid- Post-Measurement the Intervention and the Waitlist Condition

Variable	Measurement/ Condition	pre-measurement (0 weeks)	mid-measurement (8 weeks)	post-measurement (15 weeks)
		M (SD)	M (SD)	M (SD)
ECBI-I	Intervention	144.86 (18.39)	132.39 (18.65)	127.24 (19.60)
	Waitlist	143.78 (14.68)	144.31 (14.01)	136.51 (18.41)
Daily Measurements	Intervention	1.45 (0.66)	1.17 (0.59)	1.02 (0.58)
	Waitlist	1.43 (0.52)	1.30 (0.62)	1.27 (0.67)
PPI positive	Intervention	4.18 (0.54)	4.33 (0.45)	4.49 (0.57)
	Waitlist	4.32 (0.38)	4.27 (0.35)	4.34 (0.42)
PPI negative	Intervention	3.24 (0.71)	3.09 (0.59)	2.79 (0.53)
	Waitlist	3.05 (0.62)	3.16 (0.51)	3.04 (0.57)
PSOC	Intervention	62.85 (10.03)	-	66.61 (9.45)
	Waitlist	62.81 (9.43)	-	64.86 (10.29)

*ECBI-I* Eyberg Child Behavior Inventory, Intensity Scale, *PPI* Parent Practices Interview, *PSOC* Parenting Sense of Competence Scale; range ECBI-I: 36–256, range daily measurements: 0–5, range PPI: 1–7, range PSOC: 16–96

**Table 3:** Results of the Mediation Analyses

Outcome	Mediator(s)	a-path B [95% CI]	b-path B [95% CI]	Direct effect, c'-path B [95% CI]	Indirect effect, a-path*b-path B [95% CI], p	Total effect, c-path
ECBI-I	PPI positive	0.19 [0.07; 0.31]*	−2.95 [−6.90; 1.01]	−8.45 [−12.86; −4.05]*	−0.58 [−1.46; 0.29]	−10.21 [−14.62; −5.82]*
	PPI negative	−0.29 [−0.43; −0.14]*	7.14 [3.81; 10.46]*		−1.99 [−3.47; −0.51]**	
ECBI-I	PSOC	2.16 [−0.74; 5.07]	−0.48 [−0.74; −0.22]*	−9.33 [−14.59; −4.06]*	−1.04 [−2.54; 0.46]	−9.78 [−15.51; −4.04]*
Daily measurements	PPI positive	0.19 [0.07; 0.31]*	−0.11 [−0.27; 0.05]	−0.17 [−0.35; 0.003]	−0.02 [−0.06; 0.02]	−0.22 [−0.38; −0.05]*
	PPI negative	−0.29 [−0.43; −0.14]*	0.15 [0.01; 0.29]*		−0.04 [−0.10; 0.02]	
Daily measurements	PSOC	2.16 [-.74; 5.07]	−0.01 [−0.02; −0.001]*	−0.22 [−0.43; −0.02]	−0.02 [−0.06; 0.02]	−0.26 [−0.48; −0.05]*

Rows separated by lines represent separate analyses; *ECBI-I* Eyberg Child and Behavior Inventory, Intensity Scale, *PPI* Parenting Practices Interview, *PSOC* Parenting Sense of Competence Scale; * = *p* <.05, ** = *p* <.01.

## Discussion

The aim of the current study was to examine mechanisms underlying changes in child behavioral difficulties following a self-help parenting program. Our study was the first study focusing on a self-help parenting program targeting children with behavioral difficulties, with and without a diagnosis. Findings indicated that a reduction in unsupportive parenting partially mediated a reduction in parent-rated child behavioral difficulties as assessed with the ECBI-I questionnaire (19% mediation), but not when assessed with EMA (daily measurements of behavior that was targeted in the self-help program). Neither changes in supportive parenting nor parenting sense of competence mediated changes in children’s behavioral difficulties, irrespective of the type of rating (i.e., parent-report questionnaire or EMA).

The findings regarding unsupportive parenting assessed with the questionnaire align with a recent study on a self-help parenting program (Dose et al., 2021) and partially with previous studies on face-to-face parenting interventions showing that changes in unsupportive parenting mediated changes in child behavioral difficulties in approximately a quarter of the studies (Chronis-Tuscano et al., 2011; Forehand et al., 2014). However, no such mediation effect was observed when child behavioral difficulties were assessed with EMA. Several factors may explain this discrepancy between questionnaire-based versus EMA-based findings. First, the measures differ in how behavior was assessed. EMA was conducted at four consecutive weekdays (through brief telephone calls) and required the parent to report the occurrence and severity of certain behavior within the last 24 h, while the ECBI-I assessed parent-reported occurrence of certain behavior in the past two weeks. EMA is generally considered a more “objective” method that is less susceptible to recall bias and expectancy effects compared to questionnaires (Shiffman et al., 2008). It might be that using the ECBI-I, parents overestimated the impact of the intervention on their child’s behavior as the effect size for the ECBI-I (*d* = − 0.51) being slightly higher than that for EMA (*d* = 0.43). This may reflect a possible overestimation bias. Second, although the proportion of ADHD versus disruptive behavior items was approximately the same in the EMA and questionnaire, the specific items differed. For example, the ECBI-I included items related to arguments with parents or being disrespectful to parents, while the daily measurements did not, and the daily measurements included items related to having trouble waiting one’s turn, while the ECBI did not. In our study, the EMA and the ECBI-I scores correlated moderately assessed at baseline (*r* =.47), indicating that the two measures capture overlapping but distinct aspects of child behavioral difficulties, in line with previous findings (Solhan et al., 2009). To better understand these discrepancies, future (mediation) research should include both EMA (real time reports on behavior) and a questionnaire (child behavior during a longer period of time) and align specific items and metrics on both measures.

Our finding that changes in supportive parenting neither mediated changes in child behavioral difficulties – neither on the questionnaire nor on the EMA – is in line with some, though not all, studies on face-to-face parent training (Chronis-Tuscano et al., 2011; Forehand et al., 2014) as well as with the only available mediation study on self-help parent training (Dose et al., 2021). The absence of this mediation effect might partly attributable to how supportive parenting was assessed. First, the PPI did not assess all parenting practices targeted in the parenting program, such as playing with the child and providing clear instructions. In contrast, the PPI items on negative parenting were more in line with the parenting skills included in the intervention. Second, the PPI positive scale grouped different supportive parenting techniques (e.g., praising, discipline appropriately, providing clear rules), for which mediation effects could be different. To disentangle the effects of different parenting techniques, randomized micro-trials are needed (Leijten et al., 2015), in which parents are taught isolated parenting practices. Finally (Forehand et al., 2014) suggested that particularly a change from unsupportive to supportive parenting, rather than a change in either domain alone, may be the most important mediator. They therefore suggested that positive parenting may serve as a foundation for the other parenting skills learned in parent training programs, which in turn facilitate changes in child behavior.

In this study, changes in parenting sense of competence did not mediate changes in child behavioral difficulties, whereas some evidence for such mediation has been reported in studies on face-to-face parenting programs (Deković et al., 2012; Rimestad et al., 2020). To our knowledge, the current study is the first study to include parenting sense of competence as a putative mediator in a self-help parenting program. In our previous study we found that parents who choose to participate in the self-help program, already reported relatively high sense of parenting competence prior to the intervention (de Jong et al., preprint 2024) leaving limited room for improvement of parenting sense of competence and consequently for mediating effects. Furthermore, it might be that self-help parenting programs mainly focus on teaching parents behavioral techniques, with less emphasis on parental emotions and cognitions. In contrast, face-to-face programs may offer therapist support and encouragement, which might enhance parenting sense of competence more directly. This could explain the absence of a mediating effect of parenting sense of competence in our study.

Mechanisms of change could differ between face-to-face and self-help parenting programs. Firstly, because different families may enter face-to-face compared to self-help parenting programs. For example, as noted earlier, parents participating in the self-help program already had a relatively high parenting sense of parenting before starting the intervention (de Jong et al., preprint 2024) and this might be different for parents applying for face-to-face programs. As different families may enter face-to-face compared to self-help parenting programs, and for these groups other mechanisms of change may play a role, future studies are needed to study working mechanisms for different groups of parents (e.g., using moderated mediation analyses). Secondly, differences in content and delivery between self-help and face-to-face programs may also generate different mediators. For example, therapeutic alliance is an important predictor of treatment success (Kazdin & Whitley, 2006) and may be a mediator in face-to-face programs, while in self-help programs this is unlikely. In self-help programs, engagement in the online program might mediate treatment success, although this was not convincingly found in a previous study (Wähnke et al., 2024). Future studies should compare a self-help parenting program with a face-to-face program directly.

A strength of our study was that we included an ecologically valid measure of child behavioral difficulties (EMA), in addition to the widely used questionnaire. Another strength was the inclusion of a mid-study assessment, to increase statistical power and reliability of the mediator measurement, whereas most previous studies used a pre- and post-measurement only. However, some limitations of the study should be noted. First, a limited number of putative mediators and outcome measures were examined. Given that unsupportive parenting only accounted for 19% of the improvement in child behavioral difficulties, other underlying mechanisms of change, such as improvements in the parent-child relationship, may have played a role (Maric et al., 2015). Additionally, our study did not assess additional relevant outcomes, such as child functional impairment. Second, the potential reciprocal relationship between parenting practices and child behavior as shown in previous studies (Yan et al., 2021), was not taken into account. More frequent assessments (e.g., weekly) in future studies could shed more light on the temporal ordering of changes in parenting practices and changes in child behavioral difficulties. Third, all mediator variables were parent-reported. Parents may have responded in socially desirable ways - particularly regarding unsupportive parenting - given their awareness of intervention content. Future studies should incorporate not only an ecologically valid measure for child behavioral difficulties, but also for parenting practices and cognitions. Fourth, since the sample was powered to detect main effects, it may have been underpowered to detect all mediation effects. While no power analysis methods are available for mediation analyses, the detection of a mediation effect in the current study suggests sufficient statistical power to detect some mediation effects.

In conclusion, reductions in unsupportive parenting mediated improvements in child behavioral difficulties following a self-help parenting program. However, this effect was only present when child behavioral difficulties were assessed with a widely-used parent-report questionnaire and not when measured with EMA. These findings highlight the importance of including such an ecologically valid outcome measures that may be less prone to bias and expectancy effects. Positive parenting and parenting sense of competence did not mediate the relationship between self-help parenting and child behavioral difficulties. Overall, although future studies into working mechanisms of self-help parenting programs should provide insight into reciprocal relations between parental and child factors, our study suggests that reducing unsupportive parenting practices may be a central mechanism through which self-help parenting interventions exert their effects.

## Supplementary Information

Below is the link to the electronic supplementary material.

Supplementary File 1(DOCX 25.7 KB)

Supplementary File 2(DOCX 26.4 KB)

## Data Availability

Data available on reasonable request.
