# Supplementary material for: Mechanisms of Change in a Self-Help Parenting Program for Child Behavioral Difficulties: the Role of Unsupportive Parenting
Source: Res Child Adolesc Psychopathol. 2025 Nov 13;53(12):1923–34. doi: 10.1007/s10802-025-01378-y (PMC12718222; doi:10.1007/s10802-025-01378-y)
Supplement: Supplementary file 1 — (DOCX 25.7 KB) [file 10802_2025_1378_MOESM1_ESM.docx]

**Online Resource 1**

**Items selected from the List of Target Behaviors (see (Van Den Hoofdakker et al., 2007))**

- Noncompliance
- Whining, persistently complaining, opposing, discussing
- Challenging, provoking or intentionally annoying others
- Getting angry quickly and often
- Having frequent arguments with siblings
- Having temper tantrums
- Swearing
- Hitting, pinching or kicking
- Breaking things or throwing objects
- Losing things, forgetting items, or being disorganized
- Not tidying up things
- Failing to complete tasks
- Not being able to wait for one's turn
- Being overactive or restless, talking loudly, making noise
