# Supplementary material for: Mechanisms of Change in a Self-Help Parenting Program for Child Behavioral Difficulties: the Role of Unsupportive Parenting
Source: Res Child Adolesc Psychopathol. 2025 Nov 13;53(12):1923–34. doi: 10.1007/s10802-025-01378-y (PMC12718222; doi:10.1007/s10802-025-01378-y)
Supplement: Supplementary file 2 — (DOCX 26.4 KB) [file 10802_2025_1378_MOESM2_ESM.docx]

**Online Resource 2**

**Table S1**

*Differences between the intervention and waitlist condition for child and parent outcomes (as described in our previous studies (authors, 2023)*

| **Outcome measure** | **Regression results (intervention versus waitlist)** | ***p*** | ***d*** |
| --- | --- | --- | --- |
| ECBI-I (externalizing behavior) | *B (SE)* = -9.81 (2.61) | < .001 | -0.51 |
| Daily measurements of problem behavior | *B (SE)* = -0.27 (0.11) | .011 | -0.43 |

*Note: ECBI-I = Eyberg Child and Behavior Inventory, Intensity Scale;*
